# A Comprehensive Analysis of Bone Mineral Density Changes across the Lifespan: Insights from National Surveys

**DOI:** 10.3390/nu16162804

**Published:** 2024-08-22

**Authors:** Tao Li, Guimin Huang, Dongqing Hou, Yijing Cheng, Tong Zhang, Yajun Liang, Junting Liu

**Affiliations:** 1 Child Health Big Data Research Center, Capital Institute of Pediatrics, Beijing 100020, China; socott@126.com (T.L.); guiminhuang@163.com (G.H.); dqhou@sina.com (D.H.); yijing_cheng@163.com (Y.C.); zt@chinawch.org.cn (T.Z.); 2 Department of Global Public Health, Karolinska Institutet, 17177 Stockholm, Sweden; yajun.liang@ki.se

**Keywords:** bone mineral density, peak bone mass, puberty, dual-energy X-ray absorptiometry

## Abstract

Background: There is limited research providing an overall understanding of bone mineral density (BMD) changes throughout different stages of life. This study aimed to investigate the pattern of BMD changes across childhood, adolescence, adulthood, and old age, as well as exploring the critical time of peak BMD (PBMD). Methods: Participants of three major ethnicities from National Health and Nutrition Examination Survey 1999 to 2018 were involved: 46,381 and 20,944 participants aged 8–85 years old were included in the Lumbar spine BMD (LSBMD) and femoral neck BMD (FNBMD) studies, respectively. BMD was measured using dual-energy X-ray absorptiometry. The generalized additive model was used to construct smoothed percentile curves. Results: Both males and females experienced a sharp increase in LSBMD during puberty, with females reaching their PBMD earlier than males. Females’ LSBMD remained higher than males’ before the age of approximately 50, except for Non-Hispanic Blacks. For males, LSBMD reached a plateau at around 30 years old after reaching the peak value. Females exhibited two peak points on the fitted curves, with the second PBMD occurring around 36–37 years old. Ethnic variations were observed, with Non-Hispanic Blacks displaying the highest BMD levels at all ages. Non-Hispanic Whites and Mexican Americans had lower BMD levels, with Mexican Americans generally exhibiting the lowest BMD. FNBMD reached its peak earlier than LSBMD, and males consistently had higher FNBMD than females. Conclusions: This nationally representative study contributes to the understanding of BMD changes across the lifespan, and might provide guidance for bone health interventions in different population groups.

## 1. Introduction

Bone is an important component of the human body, providing protection, support, hematopoiesis, storage, and movement functions. Bone strength, which refers to the maximum load carrying capacity of bone prior to fracture, is a key indicator used to assess bone health. It is determined by the material composition and structure of the bone. One of the most important components of bone is bone mineral, which is commonly measured as bone mineral density (BMD). Individuals with osteoporosis and fractures often have lower BMD [1], with osteoporosis defined as a BMD value that is 2.5 standard deviations (SD) or more below the mean for young adults [2]. Various factors such as sex, genetics, lifestyle, diet, physical activity, and supplement intake contribute to the accumulation and loss of bone mass [3].

However, there is limited research on the overall pattern of BMD changes across childhood, adolescence, adulthood, and old age [4,5,6]. Previous studies have not included midlife participants [6] or individuals in childhood or adolescence [4,5], and the sample sizes have often been small. Even studies with larger sample sizes often have limited participants in each age group, particularly for subgroup analyses. In addition, convenience samples have been commonly used [6], which may not accurately represent the population of the selected country or region.

The peak bone mass, which refers to the highest bone mineral mass obtained in a person’s lifetime, is an important determinant of osteoporotic fracture risk [7]. Therefore, it is crucial to determine the exact age when peak bone mineral density (PBMD) occurs in order to improve bone health. However, different studies have reported inconsistent ages for PBMD, ranging from the second to fourth decades of life. The small sample sizes and short age spans of these studies may have contributed to these discrepancies [8,9,10,11,12]. In this study, we aim to provide an overall picture of BMD changes with age in humans and explore the critical time of PBMD.

## 2. Materials and Methods

### 2.1. Study Design and Participants

Our study population was derived from the National Health and Nutrition Examination Survey (NHANES), which is conducted by the National Center for Health Statistics (NCHS) of the Centers for Disease Control and Prevention (CDC) in the United States. NHANES is a series of cross-sectional surveys that use a complex, multistage probability design to sample the civilian, noninstitutionalized population across the country. BMD measurements based on dual-energy X-ray absorptiometry (DXA) are a routine part of the examination for participants aged 8 years and older [13]. We included participants who were Non-Hispanic Whites, Non-Hispanic Blacks, or Mexican Americans, aged 8 years and older, and recruited from the 1999–2018 NHANES cycles. Participants who met any of the following criteria were excluded from the DXA examination: pregnancy, self-reported history of radiographic contrast material (barium) use in past 7 days, self-reported nuclear medicine studies in the past 3 days, weight over 300 pounds, height over 6′5″, fractured both hips, had replacements of both hips, or had pins in both hips. Participants with invalid BMD data, such as those with removable or non-removable objects, positioning problems, or movement issues during the examination, were excluded. The data used in this study are publicly available and deidentified, and the protocol numbers can be found at the CDC website (https://www.cdc.gov/nchs/nhanes/irba98.htm, accessed on 26 May 2022). The NCHS Research Ethics Review Board approved NHANES, and informed consent was obtained from all study participants. For children aged 7–17 years, both parental consent and child assent were required for participation, while parental consent alone was sufficient for children aged 0–6 years.

### 2.2. Examination of BMD

We assessed lumbar spine BMD (LSBMD) and femoral neck BMD (FNBMD) in our study. BMD measurements were obtained with a Hologic QDR-4500A fan-beam densitometer (Hologic, Inc., Bedford, MA, USA) by trained and certified radiology technologists. The participants were positioned supine on the tabletop with their feet in a neutral position and hands flat by their side. A Velcro strap was used to keep the feet stationary and together. More details about the DXA examination protocol can be found in the Body Composition Procedures Manual on the NHANES website [14]. For LSBMD, specific spine scans or whole-body scans were conducted in different NHANES surveys. We selected LSBMD data based on the availability of specific spine scan data. When specific spine scan data were available, we calculated LSBMD by summing the bone mineral content of L1–L4 and dividing it by the bone area of L1–L4. If specific spine scan data were not available, we used LSBMD data from whole-body scans. The selection process for LSBMD data are described in Table S1. FNBMD data were obtained from specific femur scans. The left hip was routinely scanned unless the participant self-reported a fractured left hip, a left hip replacement, or a pin in the left hip. The right hip was scanned in this situation.

### 2.3. Assessment of Sociodemographic Characteristics

Sociodemographic characteristics were asked by trained interviewers using Computer-Assisted Personal Interview system in language used by the respondents. Participants aged 16 years and older and emancipated minors were interviewed directly, while participants under 16 years old or those who could not answer the questions themselves completed the interview with the assistance of a proxy.

### 2.4. Statistical Analysis

We calculated sampling weights following the guidelines provided by the National Center for Health Statistics. Weighted analysis of variance (ANOVA) followed by the Bonferroni correction for pairwise comparisons was used to assess differences in average BMD among subgroups. Weighted Pearson’s Chi-squared test was used to test proportion differences among categorical factors. We used a generalized additive model (GAM) with a Gaussian family, identity link function, and smoothing spline to investigate the relationship between BMD and age and fit smoothed percentile curves. After identifying the age corresponding to the peak BMD point in each curve, we calculated the weighted mean (SD) of BMD at this age to represent the PBMD of the specific population group. We also conducted stratified analyses by sex and ethnicity. A *p*-value < 0.05 was considered statistically significant. We performed all statistical analyses using R statistical software, version 4.1.2 (R Foundation for Statistical Computing, Vienna, Austria), and we applied the R package mgcv for fitting the GAM.

## 3. Results

### 3.1. General Characteristics of the Population

**Figure 1:**
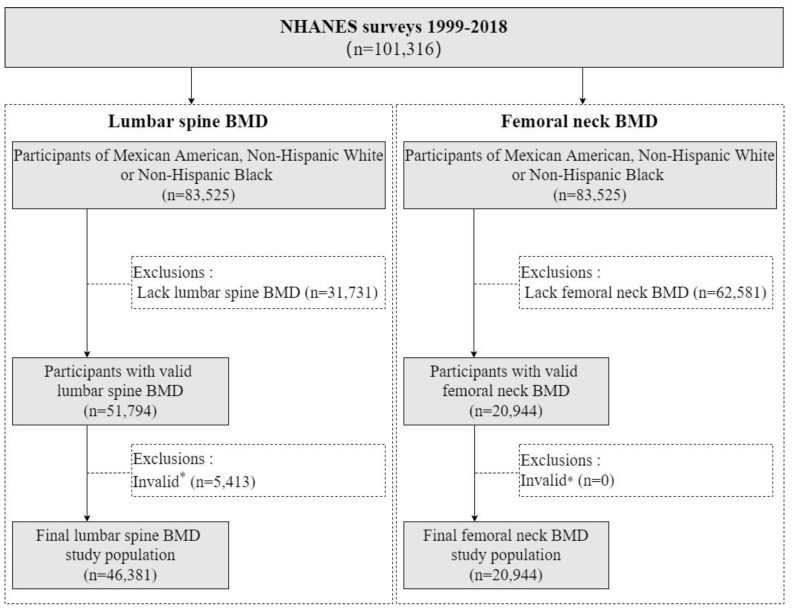
Flowchart. Abbreviations: NHANES, National Health and Nutrition Examination Survey; BMD, bone mineral density. * Invalid: The participants with removable or non-removable objects, positioning problems, movement and other problems when BMD was examined.

In the LSBMD study, we excluded 31,731 participants due to lack of LSBMD data and 5413 participants due to invalid BMD. In the FNBMD study, we excluded 62,581 participants due to lack of FNBMD data, but no participants were excluded due to BMD examination problems. Ultimately, we included a total of 46,381 participants for the LSBMD study and 20,944 participants for the FNBMD study, aged 8–85 years old (Figure 1). Among the LSBMD study participants, 52.1% (n = 24,160) were male, and the weighted proportions of Mexican Americans, Non-Hispanic Whites, and Non-Hispanic Blacks were 12.2%, 74.9%, and 13.0%, respectively. Non-Hispanic Whites had a higher average age and education level compared to the other ethnic groups (*p* < 0.001 for both). In the FNBMD study, 51.8% (n = 10,848) were male, and the weighted proportions of Mexican Americans, Non-Hispanic Whites, and Non-Hispanic Blacks were 10.5%, 77.8%, and 11.7%, respectively. Non-Hispanic Whites had a higher average age and education level, followed by Non-Hispanic Blacks and Mexican Americans (*p* < 0.001 for both, Table 1).

### 3.2. Age-Related Changes in LSBMD Levels and PBMD and Corresponding Ages, Stratified by Sex

**Figure 2:**
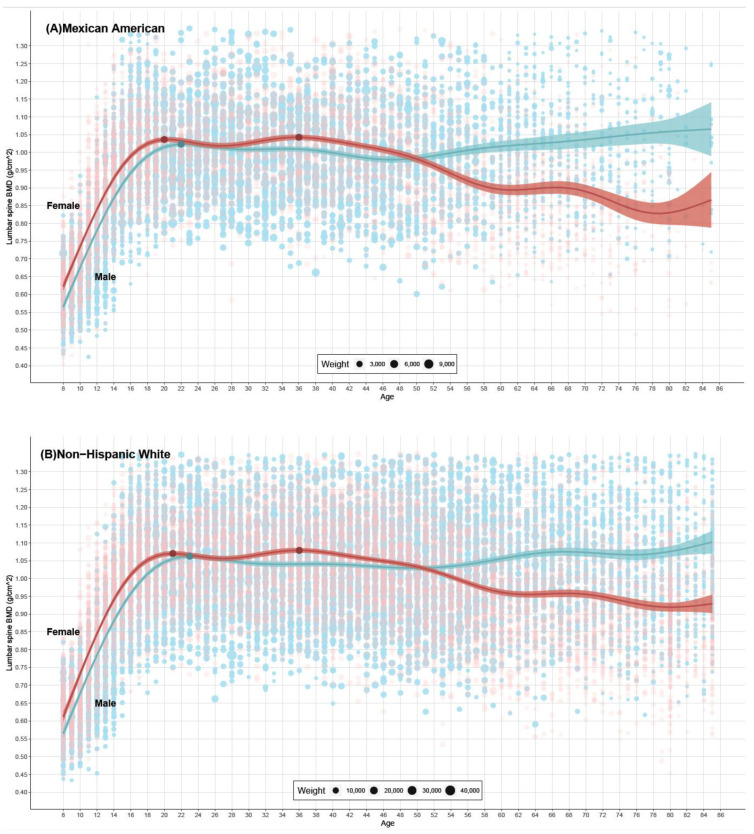

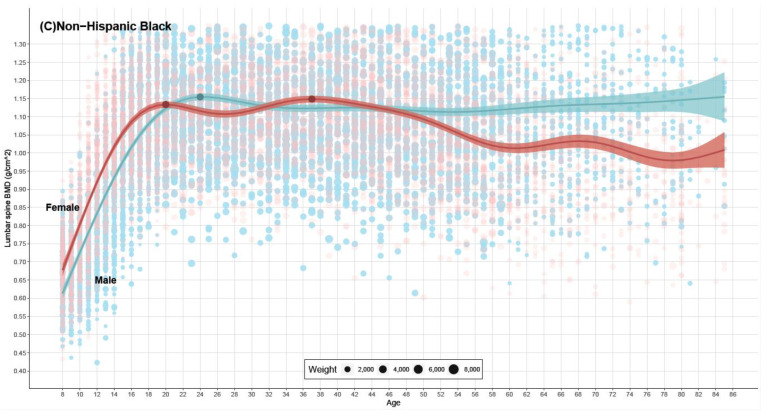
Lumbar spine BMD for different sex, stratified by ethnicity, from NHANES 1999–2018. Note: Red represents females, and blue represents males. The dark points on the lines represent the peak bone mineral density (PBMD). The light-colored points represent the participants in the survey, and the size of the points indicates the weight of that participant in the data analysis of this study. In the legend of (**A**–C), the smallest point represents 3000, 10,000 and 2000 individuals respectively.

In general, both males’ and females’ LSBMD increased sharply before reaching the lumbar spine PBMD, which typically occurred around the first half of the third decade of life. Females reached their PBMD earlier than males. Before the age of around 50, females had higher LSBMD levels compared to males among Mexican Americans and Non-Hispanic Whites, but this trend did not continue for Non-Hispanic Blacks, where females’ LSBMD levels plateaued after reaching their PBMD. In males, LSBMD slightly decreased after reaching the peak value and then reached a plateau at around 30 years old. This plateau lasted for approximately 20 years before a slight increase was observed at around 50 years old (Figure 2).

For females, two peak points were found in the fitted curves, resulting in two lumbar spine PBMD values. Males reached their lumbar spine PBMD at around 22–24 years old, which was later than females’ first PBMD at around 20–22 years old. Females reached their second PBMD at around 36–37 years old, and the corresponding BMD values were slightly higher than the first PBMD, although these differences were not statistically significant.

### 3.3. Comparison of LSBMD Levels among Different Ethnic Groups

Overall, the three ethnic groups exhibited similar patterns of fitted curves for both males and females, with Non-Hispanic Blacks consistently having the highest BMD levels at all ages, followed by Non-Hispanic Whites and Mexican Americans (Figure S1). Before the age of approximately 14, Non-Hispanic Whites and Mexican Americans had similar LSBMD levels, which were both significantly lower than those of Non-Hispanic Blacks. Non-Hispanic Blacks had the highest PBMD values in both males and females, followed by Non-Hispanic Whites and Mexican Americans (Table 2).

### 3.4. Age-Related Changes in FNBMD Levels and PBMD and Corresponding Ages, Stratified by Sex

**Figure 3:**
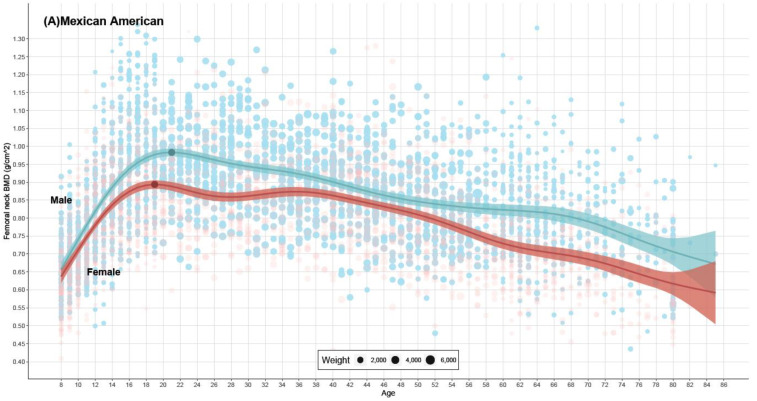

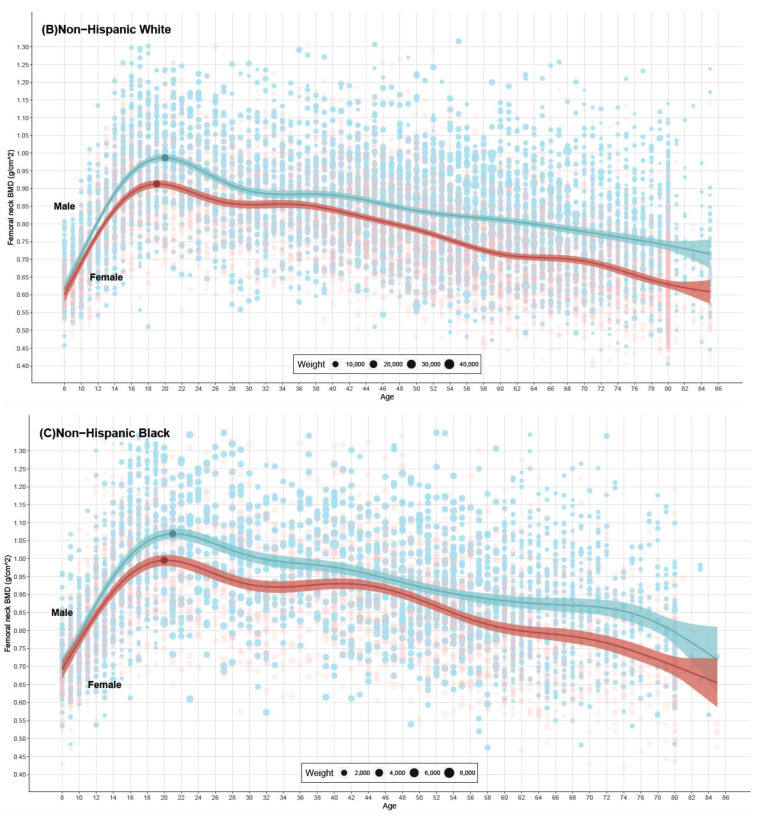
Femoral neck BMD for different sexes, stratified by ethnicity, from NHANES 1999–2018. Note: Red represents females, and blue represents males. The dark points on the lines represent the peak bone mineral density (PBMD). The light-colored points represent the participants in the survey, and the size of the points indicates the weight of that participant in the data analysis of this study. In the legend of (**A**–**C**), the smallest point represents 2000, 10,000 and 2000 individuals respectively.

FNBMD reached its peak value earlier than LSBMD, typically around the age of 19–21. Before reaching the PBMD, both males’ and females’ FNBMD increased continuously. Males’ FNBMD was higher than females’ at all ages and peaked later than females for all ethnic groups (Figure 3). After reaching the PBMD, females’ FNBMD started to decrease and then reached a plateau between the ages of 26 and 36 for Mexican Americans and Non-Hispanic Whites, and between the ages of 32 and 42 for Non-Hispanic Blacks. Females reached their femoral neck PBMD 1–2 years earlier than males, with smaller age differences compared to lumbar spine PBMD (2–4 years).

### 3.5. Comparison of FNBMD Levels among Different Ethnic Groups

Non-Hispanic Blacks consistently had the highest FNBMD levels at all ages and the highest PBMD values in both males and females (*p* < 0.05 for all). The differences in BMD values between Mexican Americans and Non-Hispanic Whites were smaller compared to those observed for lumbar spine BMD (Figure S2).

## 4. Discussion

This study provides a comprehensive overview of changes in lumbar spine and femoral neck BMD and the critical time of PBMD. We found that LSBMD and FNBMD increase significantly during puberty, highlighting the importance of this period for bone health [6,15]. However, routine BMD examinations are not recommended for the general childhood and adolescent population, as indicated by the American Academy of Pediatrics (AAP). Instead, BMD scanning should be determined by a doctor based on the child’s clinical situation [16]. It is recommended for children and adolescents with specific clinical conditions, such as recurrent fractures, bone pain, deformities, or BMD lower than age-specific standards, or those with specific diseases such as cystic fibrosis and childhood cancer.

After reaching the peak BMD, females’ BMD decreases at both sites due to a sharp decrease in estrogen concentration in the body [3]. Interestingly, we observed a slight increase in LSBMD among males after the age of 50, which is supported by another study reporting an increase of 0.4% per year in lumbar spine BMD among males aged 50 and older [5]. This suggests that males may experience a renewed period of bone accumulation after a long plateau phase. However, it should be noted that this increase in LSBMD among older males may be attributed to degenerative diseases of the lumbar spine or abnormal calcification in the surrounding tissues [17,18,19,20]. Liu’s study showed that osteophytes were more common in males in the lumbar spine compared to those in females at the hip, which substantially influenced LSBMD measurements made by DXA in the anteroposterior position on subjects over the age of 60 years [19]. However, another study conducted in Japan found that degenerative spinal diseases were common among females aged 60 and older as well [20]. The discrepancy between males and females may be due to the impact of menopause on females, as decreased estrogen levels lead to higher bone turnover [3]. Compared with the increase in BMD caused by degenerative diseases, the decrease in estrogen had a greater effect on the decrease in BMD. In a study, no increasing trend was found among Korean males [21], whereas among Han Chinese men over 50 years old, a slight increase but no statistical significance was found [22]. It is worth noting that hip and femoral BMD are more commonly recommended for diagnosing osteoporosis in the elderly, as the agreement in classifying skeletal status between spine and femoral neck BMD is only around 67% [17].

BMD is influenced by various factors, including genetic variations, dietary habits, and lifestyle choices [3,23]. Non-Hispanic Blacks consistently had the highest BMD levels among all ages and in both males and females. Mexican American and Non-Hispanic White females had higher LSBMD levels before menopause and achieved higher lumbar spine PBMD than males. But in Nguyen’s study, females’ LSBMD was slightly higher than males’ during puberty [15]. However, Non-Hispanic Black females had lower LSBMD levels at 20–30 years old and achieved lower PBMD than males. Furthermore, while males consistently had higher FNBMD levels than females at all ages [21]. Physical activity is a significant factor that contributed to the accumulation and loss of bone mass, in addition to genetic factors [24,25,26]. Under general physical expenditure in normal life and work, the association between LSBMD and physical activity was not as strong as that found between FNBMD and physical activity. A study in England showed that males spent more time in moderate-to-vigorous-intensity activity and had greater adult FNBMD compared with females [27]. The same result was also found is US, in which higher intensive physical activity was significantly associated with higher femoral BMD [28]. There are different conclusions for different genders. In a cohort study, weight-bearing physical activity frequency in the past was positively associated with hip BMD in elderly women but not in men [29]. However, when all leisure time and physical activity were considered, the association was stronger in men than women [30]. However, the association between LSBMD and physical activity is not as strong. LSBMD was only associated with higher intensive activity among males [28,31].

The age at which PBMD occurs is earlier in females than in males; however, different studies have reported inconsistent ages for PBMD, ranging from the second to fourth decades of life [4,6,32,33,34,35,36,37,38]. These discrepancies may be attributed to the small sample sizes, short age spans, and statistical analysis methods used in previous studies. In most of the studies, the study populations always were with small sample size which made them impossible to set original age data as an independent variable for curve fitting, and the study populations were usually artificially divided into certain age groups of 5 or 10 years for analysis. Then, the largest BMD mean of these groups was determined as PBMD, and the mean age or group age range was determined as the corresponding age of PBMD [12,21]. In these situations, the age grouping points partly determined the result. In addition, the different DXA methodology may also affect the results [39]. In our study, we aim to provide a more comprehensive understanding of PBMD by utilizing a larger sample size and considering different demographic factors. Additionally, we used a GAM to model the bone mineral curves, allowing for non-linear relationships between age and BMD. Our study is the first to provide nationally representative data on BMD across different age groups, including childhood, adolescence, adulthood, and old age. There are several limitations in our study. First, LSBMD data from different sources (whole body scan or specific spine scan) may affect the accuracy of curve fitting. However, studies have shown that there is no significant difference between these two data scans [40]. Second, this study uses data from cross-sectional surveys to represent longitudinal changes with age. Cross-sectional studies may overestimate the rate of bone mass loss compared with longitudinal studies [4,41].

## 5. Conclusions

In conclusion, our study confirms that bone mineral mainly accumulates during puberty. Lumbar spine BMD reaches its peak during the first half of the second decade of life, which is later than the peak of femoral neck BMD. Females typically reach their peak BMD earlier than males, with variations observed among different ethnic groups and regions. Further research based on longitudinal studies is needed to validate these findings.

## Figures and Tables

**Table 1:** Weighted characteristics of the study population by sex and ethnicity.

Characteristics	Participants, No. (Weighted %)					
	Male			Female		
	Mexican American	Non-Hispanic Whites	Non-Hispanic Blacks	Mexican American	Non-Hispanic Whites	Non-Hispanic Blacks
Lumbar spine BMD analysis (N = 46,381) ^#^	6509 (12.2)	10,869 (74.9)	6782 (13.0)	5850 (10.4)	10,077 (75.5)	6294 (14.1)
Age (years)						
8–12 (childhood)	1255 (12.9)	1256 (8.2)	1252 (11.9)	1099 (14.1)	1078 (7.1)	1143 (10.3)
13–18 (adolescence)	1623 (14.2)	1614 (10.5)	1706 (14.7)	1295 (14.3)	1293 (9.3)	1274 (11.4)
19–44 (young adulthood)	2144 (53.9)	3844 (43.4)	2106 (45.7)	1892 (49.1)	3472 (40.1)	1972 (44.2)
45–64 (midlife)	1040 (16.4)	2445 (29.0)	1232 (22.9)	1079 (18.5)	2491 (31.3)	1405 (27.3)
65–85 (late life)	447 (2.6)	1710 (8.9)	486 (4.8)	485 (4.0)	1743 (12.2)	500 (6.8)
Education level *						
<College degree	3250 (92.2)	5775 (67.7)	3161 (84.6)	3155 (90.9)	5662 (68.2)	3159 (80.9)
≥College degree	242 (7.8)	2210 (32.3)	559 (15.4)	247 (9.1)	2077 (31.8)	691 (19.1)
Ratio of family income to poverty (mean ± SD)	1.79 ± 1.32	2.92 ± 1.64	2.14 ± 1.54	1.78 ± 1.36	2.81 ± 1.64	2.02 ± 1.51
Femoral neck BMD analysis (N = 20,944) ^#^	2581 (10.5)	5479 (77.8)	2788 (11.7)	2393 (8.7)	5119 (78.6)	2584 (12.7)
Age (years)						
8–12 (childhood)	431 (10.4)	397 (5.4)	330 (8.0)	422 (12.1)	372 (4.8)	356 (8.1)
13–18 (adolescence)	442 (9.8)	507 (7.3)	458 (10.6)	414 (11.1)	445 (6.6)	421 (9.6)
19–44 (young adulthood)	795 (47.1)	1393 (29.8)	669 (35.0)	685 (40.0)	1327 (28.6)	627 (33.8)
45–64 (midlife)	644 (25.9)	1581 (38.2)	835 (34.6)	599 (27.5)	1519 (37.4)	777 (35.1)
65–85 (late life)	269 (6.8)	1601 (19.3)	496 (11.8)	273 (9.4)	1456 (22.6)	403 (13.3)
Education level *						
<College degree	1500 (91.4)	3246 (67.2)	1619 (84.6)	1369 (90.3)	3132 (68.6)	1443 (81.4)
≥College degree	125 (8.6)	1252 (32.8)	295 (15.4)	124 (9.7)	1118 (31.4)	305 (18.6)
Ratio of family income to poverty (mean ± SD)	1.84 ± 1.36	2.96 ± 1.63	2.34 ± 1.55	1.84 ± 1.38	2.86 ± 1.63	2.17 ± 1.51

Abbreviations: BMD, bone mineral density; SD, standard deviation. ^#^ The LSBMD data were from NHANES 1999–2018, and FNBMD data were from NHANES 2005–2010, 2013–2014, and 2017–2018. * The education level data for participants < 20 years old was from household reference person. The household reference person is the first household member 18 years of age or older listed on the household member roster who owns or rents the residence where members of the household reside. Participants with missing values are excluded.

**Table 2:** PBMD values of lumbar spine and femoral neck by sex and ethnicity, and the corresponding ages.

Sex and Bone Sites	PBMD, Weighted Mean ± SD, g/cm^2^			Corresponding Age (Weighted No. *), Mean, Years		
	Mexican American	Non-Hispanic Whites	Non-Hispanic Blacks	Mexican American	Non-Hispanic Whites	Non-Hispanic Blacks
Lumbar spine						
Male	0.985 ± 0.098 ^a^	1.055 ± 0.119 ^b^	1.132 ± 0.146 ^c^	22 (n = 238,323)	23 (n = 1,117,690)	24 (n = 179,315)
Female Ⅰ ^#^	1.016 ± 0.120 ^a^	1.059 ± 0.115 ^ab^	1.095 ± 0.131 ^b^	20 (n = 202,365)	21 (n = 885,651)	20 (n = 231,616)
Female II ^#^	1.013 ± 0.105 ^a^	1.078 ± 0.131 ^b^	1.140 ± 0.154 ^c^	36 (n = 182,449)	36 (n = 999,886)	37 (n = 200,151)
*P*_sex_	0.105	0.122	0.698			
Femoral neck						
Male	0.967 ± 0.099 ^a^	0.957 ± 0.111 ^a^	1.037 ± 0.199 ^b^	21 (n = 89,648)	20 (n = 361,998)	21 (n = 75,213)
Female	0.881 ± 0.134 ^a^	0.893 ± 0.111 ^a^	1.000 ± 0.152 ^b^	19 (n = 53,746)	19 (n = 203,033)	20 (n = 66,661)
*P*_sex_	<0.001	<0.001	<0.001			

Abbreviations: PBMD, peak bone mineral density; SD, standard deviation. * Weighted No., the weighted number of the PBMD corresponding age group population. ^#^ Two PBMD were calculated since there were two numerically similar peak points on females’ LSBMD fitted curves. ^a^, ^b^, ^c^, The values with the same letter are not significantly different between different ethnicities (*p* < 0.05). Note: the PBMD (mean ± SD) values were calculated from the weighted original values of the study population, which were different from the fitted values of GAM. The GAM was just used to find the corresponding age of PBMD.

## Data Availability

All relevant data are available at https://wwwn.cdc.gov/nchs/nhanes, accessed on 15 January 2023.

## Supplementary Materials

The following supporting information can be downloaded at: https://www.mdpi.com/article/10.3390/nu16162804/s1. Table S1. The selection of lumbar spine BMD data. Figure S1. Lumbar spine BMD for different ethnicity, stratified by sex, from NHANES 1999–2018. Figure S2. Femoral neck BMD for different ethnicity, stratified by sex, from NHANES 1999–2018.
