# Supplementary material for: A Comprehensive Analysis of Bone Mineral Density Changes across the Lifespan: Insights from National Surveys
Source: Nutrients. 2024 Aug 22;16(16):2804. doi: 10.3390/nu16162804 (PMC11356834; doi:10.3390/nu16162804)
Supplement: Supplementary file 1 [file nutrients-16-02804-s001.zip › nutrients-3127788-supplementary.pdf]

Supplements

Table S1 The selection of lumbar spine BMD data

CYCLE	Lumbar	L1-L4	Final included
1999-2000	8-85	-	lumbar
2001-2002	8-85	-	lumbar
2003-2004	8-85	-	lumbar
2005-2006	8-69	8-85	L1-L4
2007-2008	-	8-80	L1-L4
2009-2010	-	8-80	L1-L4
2011-2012	8-59	-	lumbar
2013-2014	8-59	40-80	lumbar (8-39), L1-L4 (40-80)
2015-2016	8-59	-	lumbar
2017-2018	8-59	50-80	lumbar (8-49), L1-L4 (50-80)

-Not available

Figure S1 Lumbar spine BMD for different ethnicity, stratified by sex, from NHANES 1999-2018.

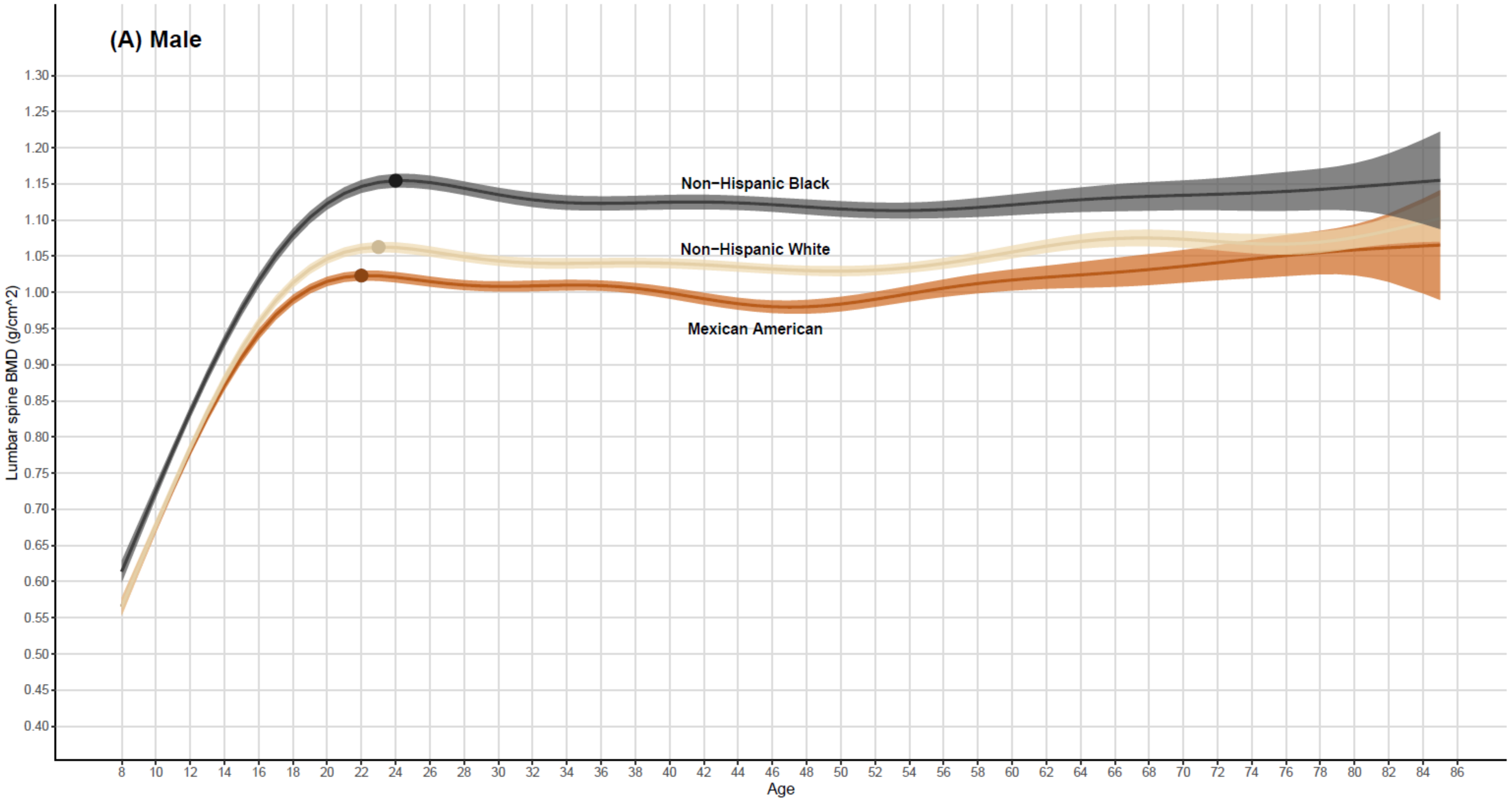

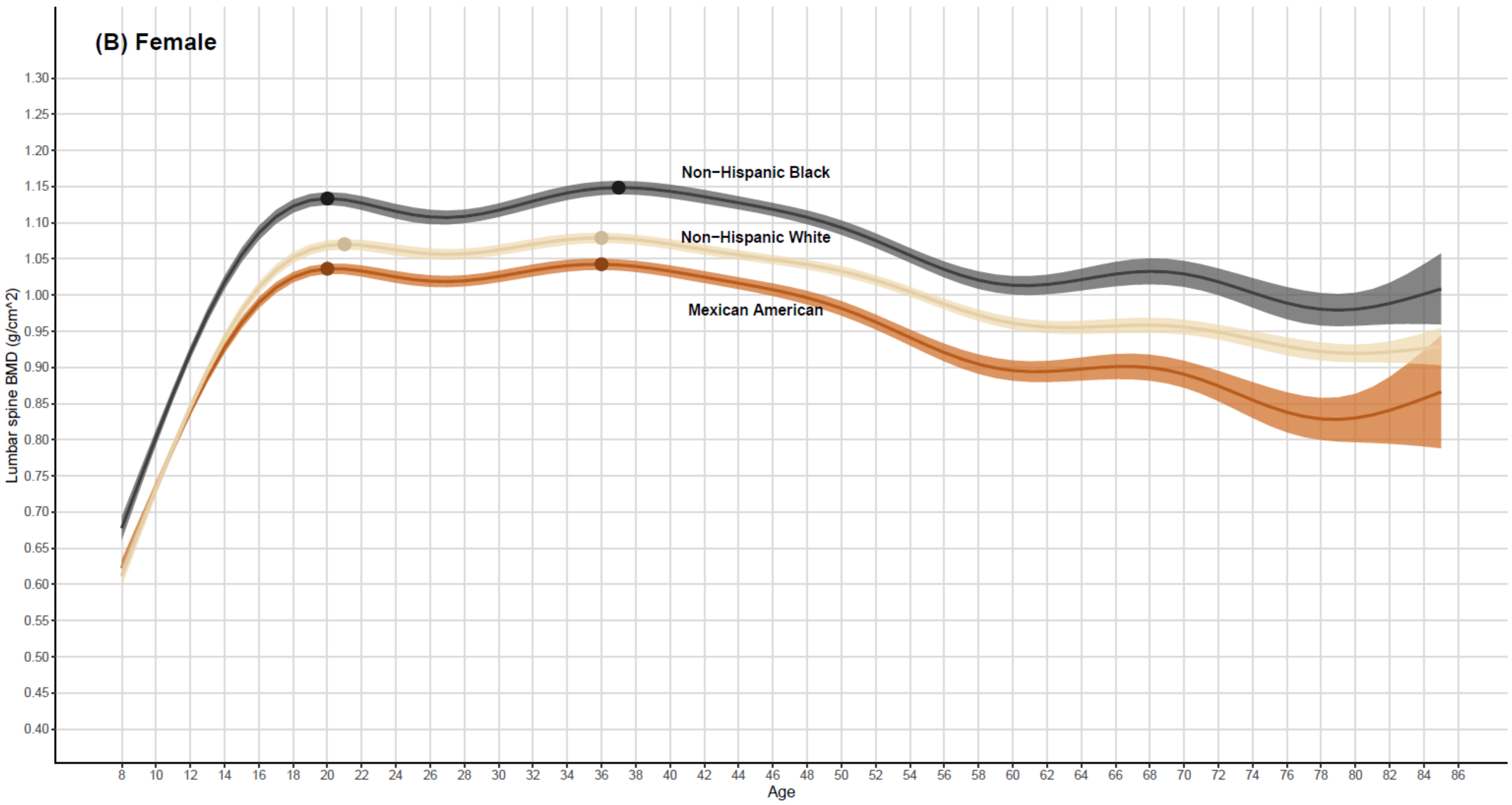

(B) Female
Non−Hispanic Black
Non−Hispanic White
Mexican American
Lumbar spine BMD (g/cm^2)
1.30
1.25
1.20
1.15
1.10
1.05
1.00
0.95
0.90
0.85
0.80
0.75
0.70
0.65
0.60
0.55
0.50
0.45
0.40
8 10 12 14 16 18 20 22 24 26 28 30 32 34 36 38 40 42 44 46 48 50 52 54 56 58 60 62 64 66 68 70 72 74 76 78 80 82 84 86
Age

Figure S2 Femoral neck BMD for different ethnicity, stratified by sex, from NHANES 1999-2018.

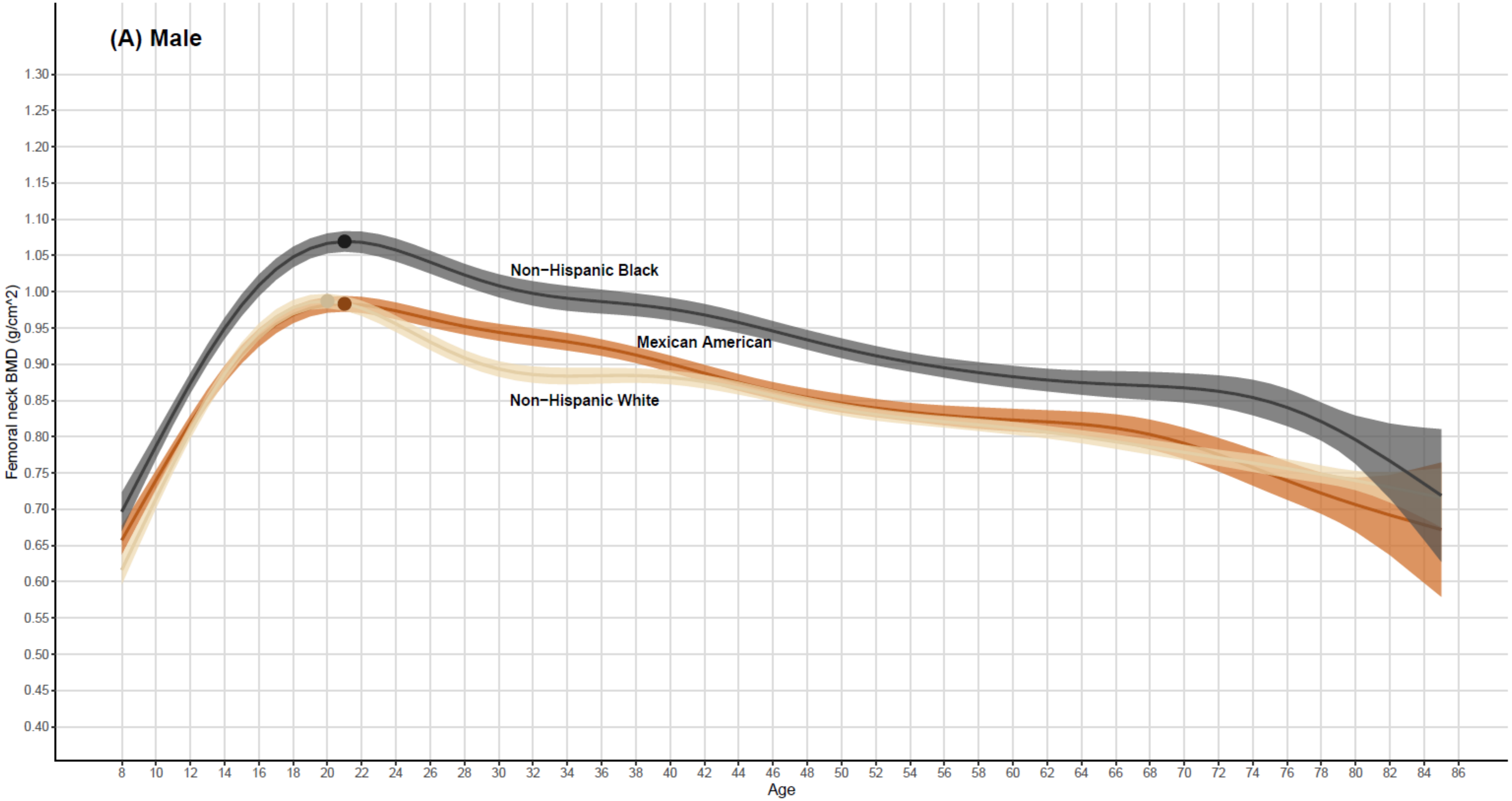

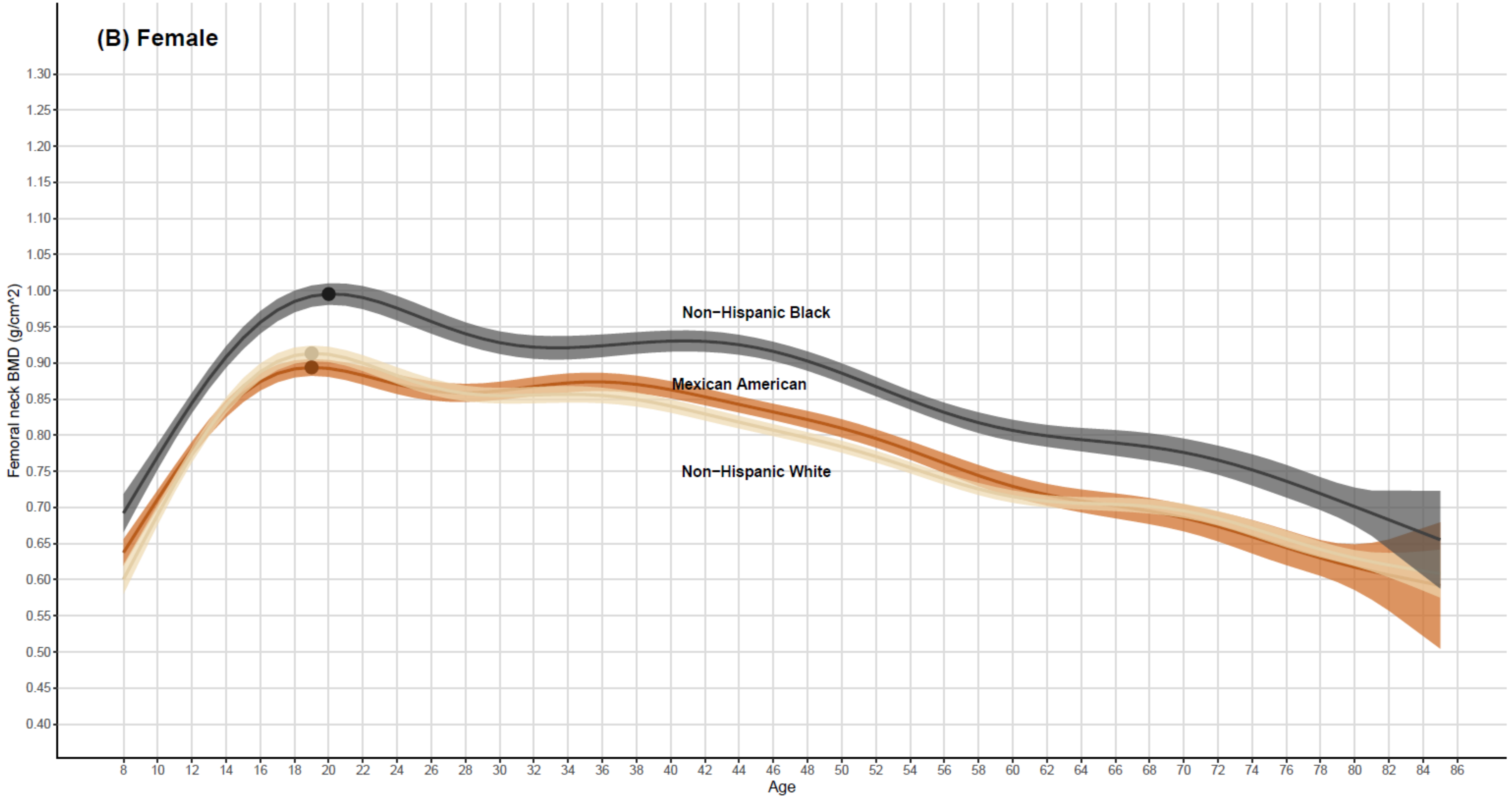
(B) Female
Non−Hispanic Black
Mexican American
Non−Hispanic White
Femoral neck BMD (g/cm^2)
Age
1.30
1.25
1.20
1.15
1.10
1.05
1.00
0.95
0.90
0.85
0.80
0.75
0.70
0.65
0.60
0.55
0.50
0.45
0.40
8 10 12 14 16 18 20 22 24 26 28 30 32 34 36 38 40 42 44 46 48 50 52 54 56 58 60 62 64 66 68 70 72 74 76 78 80 82 84 86
